# Thermal fluctuations affect the transcriptome through mechanisms independent of average temperature

**DOI:** 10.1038/srep30975

**Published:** 2016-08-04

**Authors:** Jesper Givskov Sørensen, Mads Fristrup Schou, Torsten Nygaard Kristensen, Volker Loeschcke

**Affiliations:** 1 Department of Bioscience, Section for Genetics, Ecology and Evolution, Aarhus University, Ny Munkegade 114, 8000 Aarhus C, Denmark; 2 Department of Chemistry and Bioscience, Aalborg University, Fredrik Bajers Vej 7H, 9220 Aalborg E, Denmark

## Abstract

Terrestrial ectotherms are challenged by variation in both mean and variance of temperature. Phenotypic plasticity (thermal acclimation) might mitigate adverse effects, however, we lack a fundamental understanding of the molecular mechanisms of thermal acclimation and how they are affected by fluctuating temperature. Here we investigated the effect of thermal acclimation in *Drosophila melanogaster* on critical thermal maxima (CTmax) and associated global gene expression profiles as induced by two constant and two ecologically relevant (non-stressful) diurnally fluctuating temperature regimes. Both mean and fluctuation of temperature contributed to thermal acclimation and affected the transcriptome. The transcriptomic response to mean temperatures comprised modification of a major part of the transcriptome, while the response to fluctuations affected a much smaller set of genes, which was highly independent of both the response to a change in mean temperature and to the classic heat shock response. Although the independent transcriptional effects caused by fluctuations were relatively small, they are likely to contribute to our understanding of thermal adaptation. We provide evidence that environmental sensing, particularly phototransduction, is a central mechanism underlying the regulation of thermal acclimation to fluctuating temperatures. Thus, genes and pathways involved in phototransduction are likely of importance in fluctuating climates.

It is widely accepted that global climate change will affect ectothermic organisms through changes in means and variances of temperatures^1^. However, exactly how organisms will be affected is less well understood^2^. It is, for example, not clear whether tropical or mid-latitude terrestrial ectotherms are most vulnerable to warming^3^,^4^. Recent studies points to a major impact of extreme events rather than mean temperatures on the distribution of populations^5^,^6^,^7^, and it has been argued that many species are likely to depend heavily on adaptive behavioral or physiological responses (acclimation) to survive variable and sometimes stressful thermal conditions^8^,^9^,^10^. Studying in depth if, when and how organisms respond to temperature challenges through modifications of physiology or behavior is therefore important for a better understanding of thermal adaptation in general and the effects of thermal variation specifically^11^,^12^.

From laboratory studies of ectotherms we have obtained detailed information about costs and benefits of acclimation at low and high temperatures^8^,^12^. Generally, beneficial thermal acclimation is known to affect thermal preference and performance, including increasing tolerance to subsequent exposure to more stressful temperatures^8^,^13^. In nature terrestrial ectotherms are rarely exposed to constant thermal conditions, however, current knowledge of temperature acclimation relies primarily on studies at constant temperatures^14^. Hardening and acclimation at variable temperatures have been shown to induce increased heat tolerance^15^,^16^ and might elicit a different stress response to subsequent temperature challenges compared to a pretreatment at constant temperatures^17^,^18^,^19^. Furthermore, the few studies investigating plastic responses to low and high temperatures in the field or under semi-natural conditions do not always support conclusions based on laboratory studies as costs and benefits may differ in these two very different environments^20^,^21^. Thus, knowledge on the physiological mechanisms affected by mean and natural fluctuations in temperature is crucial for understanding adaptation to varying climatic conditions from hourly to annual scales^19^.

Global or targeted molecular studies have identified and investigated the physiological mechanisms underlying responses to short term exposure to cold or hot pretreatments^22^,^23^,^24^,^25^,^26^,^27^ and the functional basis for increased heat and cold resistance^28^,^29^,^30^. However, while developmental temperature strongly impacts on adult heat tolerance we have little information about the molecular mechanisms through which thermal acclimation mediates heat tolerance in adult insects^10^,^23^. Further, we lack knowledge on the molecular mechanisms by which thermal fluctuations affect heat tolerance (but see Podrabsky & Somero)^18^. Thus, it is not only relevant to consider the relative contribution of plastic and genetic adaptation to thermal stress^31^,^32^, but also how different components of the thermal environment, including ecologically relevant fluctuating thermal conditions during development, influence the molecular physiology and thus contribute to changes in heat tolerance.

In this study we investigate the acclimation of a Danish population of *D. melanogaster* to mean and fluctuation of temperature at the transcriptomic level, and attempt to discern whether corresponding acclimation effects on heat tolerance are mediated by independent or shared molecular mechanisms and pathways. We submitted flies to four thermal treatments during development and early adult-life, constant temperatures of either 15 or 25 °C or fluctuating temperatures with mean temperatures of 15 or 25 °C. Using a dynamic thermal tolerance assay^33^ we measured high temperature tolerance of a set of adult flies from each of the four thermal treatments. In parallel, we performed global gene expression analyses on another set of flies from each of the four thermal treatments (shortly acclimated to 20 °C) and on flies ramped to 35 °C using the methodology of the thermal tolerance assay (see Fig. 1 for design). With this design we aimed at investigating three hypotheses at the transcriptomic and/or phenotypic levels: 1) Thermal fluctuations induce transcriptional responses that overlap with the responses to mean temperature acclimation (long term) or to the induced heat shock response during thermal ramping (short term). 2) The effect size of the response to thermal fluctuations is small, i.e. it has little effect on heat tolerance and the transcriptome, compared to the response to mean temperature. 3) Regarding heat tolerance, the effect of thermal fluctuations will depend on the mean temperature, i.e. the phenotypic change is primarily controlled by the absolute temperatures experienced and not the change in temperature itself. Similarly, the transcriptomic response to fluctuations will primarily depend on mean temperature, even though the transcriptome might also reveal a common response driven by the fluctuation itself. Our design enabled us to generate knowledge on the impact of ecologically relevant thermal fluctuation (Figure S1) at both the organismal tolerance and molecular level thereby broadening the understanding of plastic adaptation to a variable climate.

## Results

### Heat tolerance phenotype

Both mean temperature and temperature fluctuations had a marked positive effect on heat tolerance (Fig. 2). CTmax was lowest for flies from constant 15 °C and highest (with an absolute difference of about 0.75 °C) for flies from the warmer (25 °C) and fluctuating treatment. Two-way ANOVA showed a highly significant effect of mean temperature (15 or 25 °C) (F_(1,189)_ = 67.0, P < 0.001) and temperature regime (constant or fluctuating) (F_(1,189)_ = 9.7, P = 0.002), while the interaction among these two factors was non-significant (F_(1,189)_ = 3.4, P = 0.067).

### Gene expression

Across treatment contrasts, a variable number of up- and down regulated genes was detected by this study (Fig. 3). The resulting lists of differentially expressed genes in each individual contrast (e.g. gene expression of flies from constant 15 and 25 °C) and Venn diagrams of the overlaps among individual contrasts within the main treatment comparisons are available in Tables S1, S2 and Figure S2. Mean temperature (15 *vs.* 25 °C) had the largest effect on the number of genes, followed by thermal ramping (20 *vs.* 35 °C) while temperature regime (constant *vs*. fluctuating) had a quite small effect on gene expression (Fig. 3). The magnitude of differential expression followed a slightly different pattern as ramping led to the largest fold change in expression followed by mean temperature, while thermal fluctuations only led to very modest fold changes (Figure S3).

Principal component analysis of the global gene expression profiles clearly separated the samples according to mean temperature along PC1 and according to heat ramping along PC2 (Fig. 4). Three-way ANOVA of the PC scores showed significant effects occurring for the first 4 PCs (Table 1). In addition to the significant effects of mean temperature and adult thermal treatment (heat ramping), we also found a significant effect of temperature fluctuation for PC1 and PC4. Thus, even though only few genes were significantly differentially expressed by the temperature fluctuation, a significant signal was detectable from this treatment.

We applied GO-term enrichment analyses (see Table S3) to identify groups of differentially expressed genes. To further investigate the functional significance of the changes in global gene expression we used GAGE^34^ to identify KEGG pathways that were significantly enriched by the treatments of the study (Fig. 5). The response to mean temperature was to a large degree common among nested treatments and was largely dominated by responses in processes related to energy production (down in 25 °C), metabolism and bio-synthesis (up in 25 °C). The response to fluctuating temperatures was more variable among treatments, possibly due to the (order of magnitude) lower number of genes. Analysis showed never-the-less a consistent up-regulation in the phototransduction pathway in flies from fluctuating environments, and a less consistent down-regulation of various metabolic and genetic processing (e.g. transcription, translation) pathways (Fig. 5B). Ramping the flies to 35 °C led to few significant KEGG pathways, most pronounced a strong up-regulation of pathways of protein processing.

### Gene expression correlations with thermal tolerance phenotype

For a high number of genes the expression was significantly correlated with the thermal tolerance phenotype of flies from the corresponding treatment. Thus, treatments that affected heat tolerance also affected mean gene expression in a specific way. We found 2717 genes (1223 up and 1494 down) at 20 °C (no heat ramping) and 2469 genes (1300 up and 1169 down) at 35 °C (heat ramping) correlated to the thermal tolerance phenotype (P < 0.001). Of these genes 1399 were shared among the two temperatures (Table S4). These shared genes showed consistent up- or down-regulation overall, with 564 up, 828 down and only seven genes correlated negatively at 20 and positively at 35 °C (Chorion protein 18, Cold shock domain-containing protein CG9705, Dmel_CG6511, Dmel_CG9293, Hsp27, Hsp90 co-chaperone Cdc37, Protein OPI10 homolog). Of the genes that correlated with the thermal tolerance phenotype at 20 °C (P < 0.001) 214 were not correlated at 35 °C (P > 0.05), while for the genes correlated at 35 °C (P < 0.001) 237 were not correlated at 20 °C (P > 0.05). Functional GO-term enrichment analyses were performed to identify the functional significance of the differentially expressed genes identified (Table 2, complete list in Table S4). The analyses of these different gene groups showed a quite clear, but distinct pattern. Thus, genes positively correlated at both treatment temperatures (i.e. gene expression in treatments leading to increased expression at both temperatures were associated with high heat tolerance) were strongly enriched for processes related to detection of light and phototransduction (Table 2). Genes positively correlated at high temperature, but negatively correlated without the heat ramp to 35 °C (i.e. responding strongly during the heat ramping) were largely dominated by respiration, energy production and mitochondrial membrane function. Finally, genes only correlated after ramping to 35 °C were dominated by typical chaperone function, such as protein folding, and by processes associated to the shut-down of normal cell function during a stress response (i.e. mRNA metabolism and regulation of gene expression).

## Discussion

### Effects of thermal regimes on thermal tolerance

**Figure 1:**
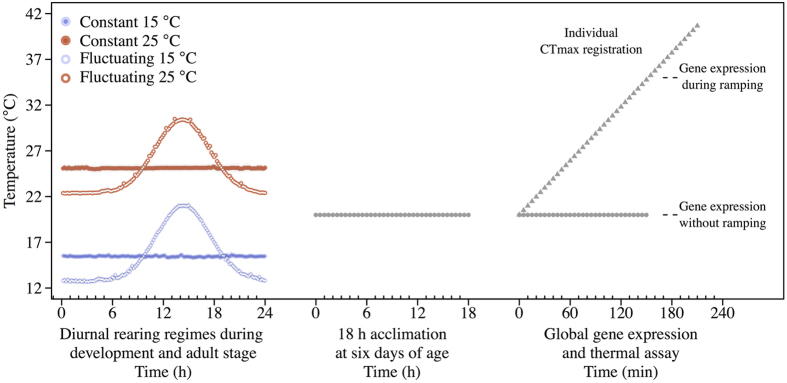
Schematic representation of the experimental design. Throughout development, flies were kept at one of the four thermal regimes (15 °C constant, 15 °C fluctuating, 25 °C constant or 25 °C fluctuating). Figure shows one cycle of 24 hours from the four distinct developmental thermal regimes, with average temperature data (±SEM) logged during the entire experimental acclimation. Prior to the heat tolerance assay all flies were moved to a common intermediate temperature of 20 °C for 18 hours. Hereafter thermal tolerance (CTmax) was estimated by increasing the temperature from 20 °C at a rate of 0.1 °C min^−1^ until immobilization of each individual fly. For microarray analyses, flies from each developmental regime were split in two groups and maintained at 20 °C or ramped from 20 °C at a rate of 0.1 °C min^−1^ until 35 °C (mean ± s.d. of realized rates in the experiments were 0.098 ± 0.001 °C min^−1^) where flies were snap frozen in liquid nitrogen.

**Figure 2:**
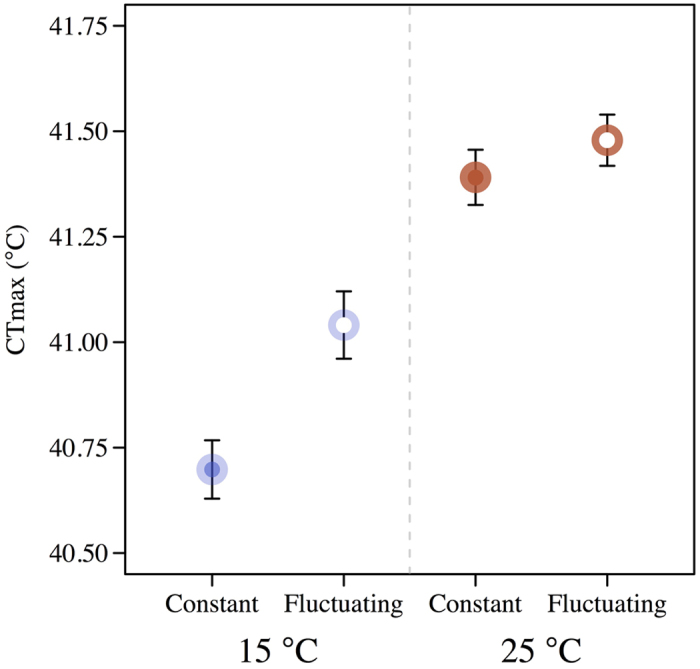
Heat tolerance phenotype assayed in a heat ramping assay. The temperature was increased from 20 °C at a rate of 0.1 °C min^−1^ until total immobilization (CTmax). Filled and open symbols represent development at constant (15 °C: n = 50; 25 °C: n = 48) and fluctuating temperatures (15 °C: n = 47; 25 °C: n = 48), respectively. Blue symbols represent development at 15 °C and red symbols development at 25 °C. Two-way ANOVA revealed significant effect of average temperature (P < 0.001) and thermal regime (P = 0.002). No post-hoc comparison was made as the interaction between thermal regime and average temperature was non-significant (P = 0.067).

In this study we investigated the effects of exposing flies from a Danish population to constant *vs*. simulated natural fluctuating temperatures (Fig. 1, Figure S1). As expected, heat tolerance of flies acclimated at 25 °C was higher compared to 15 °C acclimated flies (Fig. 2). Although the increase in thermal tolerance seems limited in absolute terms (<1 °C) it was in the expected range for this trait as critical upper limits of invertebrates, including drosophilids, increase with approximately 0.1 °C for each degree of thermal acclimation^14^,^35^ which can markedly increase survival rates at high temperatures^20^,^23^. Temperature fluctuations during development also increased heat tolerance (equivalent to developmental acclimation at increased mean temperatures of 1 to 5 °C), and the effect was statistically independent of mean temperature, despite a lower absolute increase in heat tolerance at 25 °C. Thus, even though mean temperature acclimation had the largest effect, there was a significant and noticeable contribution to the heat tolerance phenotype by the fluctuation of temperature. The relative reduction at 25 °C was not caused by lack of acclimation capacity, as an even higher CTmax than observed in this study, has been obtained with the use of higher mean temperatures for *D. melanogaster*^23^,^36^. Neither is a potential difference in physiological age between treatments expected to affect the results, as CTmax shows little age effect in this and other *Drosophila* species^37^,^38^. The fluctuating regime at a mean of 15 °C reaches a maximum temperature of 20 °C, which is not high enough to induce the heat stress response^39^, yet it has as strong or stronger induction of heat tolerance compared to the fluctuating regime at a mean of 25 °C. Thus, the increased tolerance induced by fluctuations seems unrelated to the heat stress response.

A number of studies have investigated the effects of thermal fluctuation on the physiology of ectotherms^18^,^19^,^40^,^41^,^42^,^43^. In *Drosophila sp.* effects of temperature fluctuations on heat tolerance have been reported to be both beneficial^15^,^44^, and non-existent^35^. This discrepancy is probably explained by the exact experimental procedure. If fluctuations reach the boundaries of the thermal limits, organisms are temporarily stressed and directly negatively affected by the fluctuations^3^,^19^,^45^. If temperatures fluctuate within non-stressful limits, they will affect the temperature controlled metabolism^43^ as well as the assumed costs of monitoring the environment and inducing plastic responses.

### Effect of thermal regimes on gene expression

While it is well documented that developmental thermal acclimation strongly impacts adult thermal tolerance^8^,^13^, we have a limited knowledge of the molecular background contributing to this plastic change, and the knowledge we have is primarily derived from studies at constant temperatures^10^,^23^. Here we found several interesting aspects regarding physiological mechanisms affected by temperature fluctuations. The PCA showed that transcriptional responses to mean temperature, acute exposure to heat and fluctuations act through largely independent mechanisms. If heat tolerance benefits associated with temperature fluctuations were achieved by induction of the stress response, we would expect a clear overlap between effects of high temperature exposure and fluctuations, and a large benefit in terms of increased heat tolerance of the fluctuating 25 °C treatment as this treatment reaches the temperatures that activate the heat shock response in *D. melanogaster*^46^. Neither of these expectations were met. The interpretation of independence among the three main treatments was confirmed when looking at the significant KEGG pathways and the gene ontology enrichment analyses of the genes differentially expressed among the three treatments (Fig. 5, Table S3), which had little general overlap in the identified pathways and processes identified.

The PCA and the number of genes significantly differentially expressed both show that mean rearing temperature (15 or 25 °C) and heat ramping (20 °C or ramped to 35 °C) have very large effects on the transcriptional profile. In comparison, temperature fluctuations induced very few genes overall (Tables S1 and S2), but the fluctuations still had quite an impact on heat tolerance. Thus, the few genes that were affected by fluctuations are likely to be associated with mechanisms related to heat tolerance in constant *vs.* fluctuating thermal environments. In contrast to the transcriptional responses to both mean temperature and ramping, most enriched pathways identified in response to fluctuating temperatures were contrast specific. In conjunction with the low number of genes identified this makes interpretation of the role of these pathways less convincing (Fig. 5B). However, as a single exception significant up-regulation of the KEGG pathway ‘phototransduction’ was consistently found in flies from all four experimental groups from fluctuating developmental environments independent of mean temperature (Fig. 5B). Functional mutants in genes involved in phototransduction have been linked to loss of thermal preference selection of larvae^47^ and sensing of hazardous temperatures in adult *D. melanogaster*^48^. The sensitivity of TRP (Transient Receptor Potential) channels, of which some are thermally activated, could serve to detect environmental changes^49^. The role of such a response in the laboratory is unclear as the lack of spatial temperature variation makes selection of benign microhabitats redundant. Instead, we speculate that the sensory mechanisms induced by the fluctuating environment prepare the flies, and potentially many other insect species, for an increase in temperature and thereby provide a causal link to the observed increase in CTmax. In support of a role of phototransduction for thermal tolerance, genes from this pathway have previously been linked to laboratory selection for high temperature tolerance in *D. melanogaster*^50^.

The mean temperature treatments affected large parts of the transcriptome both with respect to number of significant genes and pathways and with respect to fold change. These changes comprised enrichment of processes related to energy production, corroborating recent findings^10^. There was a large overlap in both genes and enriched physiological processes among the four individual comparisons. Thus, the same genes seemed to be affected whether flies were kept at 20 °C or ramped to 35 °C and independent of temperature fluctuations. The mean temperature affected strongly amino acid-, lipid- and carbohydrate metabolism, with increased metabolism at the higher temperature (Fig. 5A, Table S3). The oxidative phosphorylation pathway and genes related to energy production showed up-regulation in flies at 15 °C (Fig. 5A, Table S3). As we have no reason to believe that metabolism and higher energy production is causally associated with a decreased high temperature tolerance, this response might rather constitute a compensatory response to maintain appropriate energy regulation and a direct effect of temperature on the metabolic activity in flies from 25 °C (Fig. 5A).

**Figure 3:**
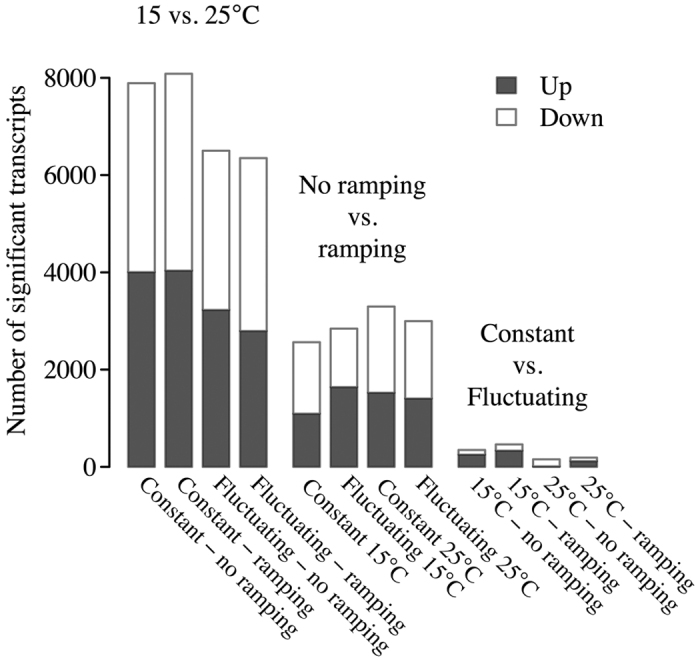
Barplot of significant of up- and down regulated genes in individual contrasts. Mean developmental temperature (15 vs 25 °C) had the largest effect on the number of genes differentially expressed, thermal high temperature ramping had an intermediate effect and temperature regime (constant *vs*. fluctuating) had a small effect on gene expression.

**Figure 4:**
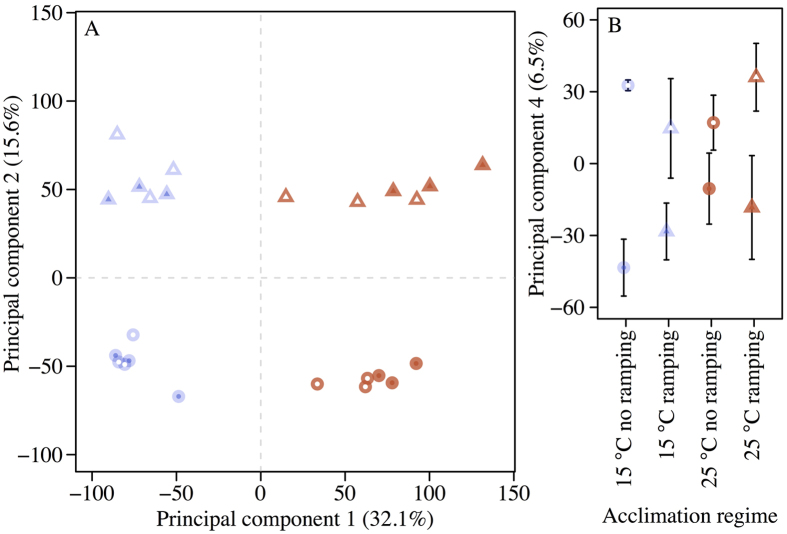
Principal component (PC) analysis of gene expression profiles. Each point represents the data from one biological replicate (one microarray). Open and closed symbols represent fluctuating and constant temperatures, respectively. Circles represent 20 °C temperature treatment and triangles represent 35 °C heat ramping treatment before sampling. Developmental temperatures are represented by blue (development at a mean of 15 °C) or red symbols (development at a mean of 25 °C), respectively. (**A**) The first two PC axes. (**B**) The score of the fourth PC showing the effect of thermal fluctuations (see Table 1 for the statistical results).

**Figure 5:**
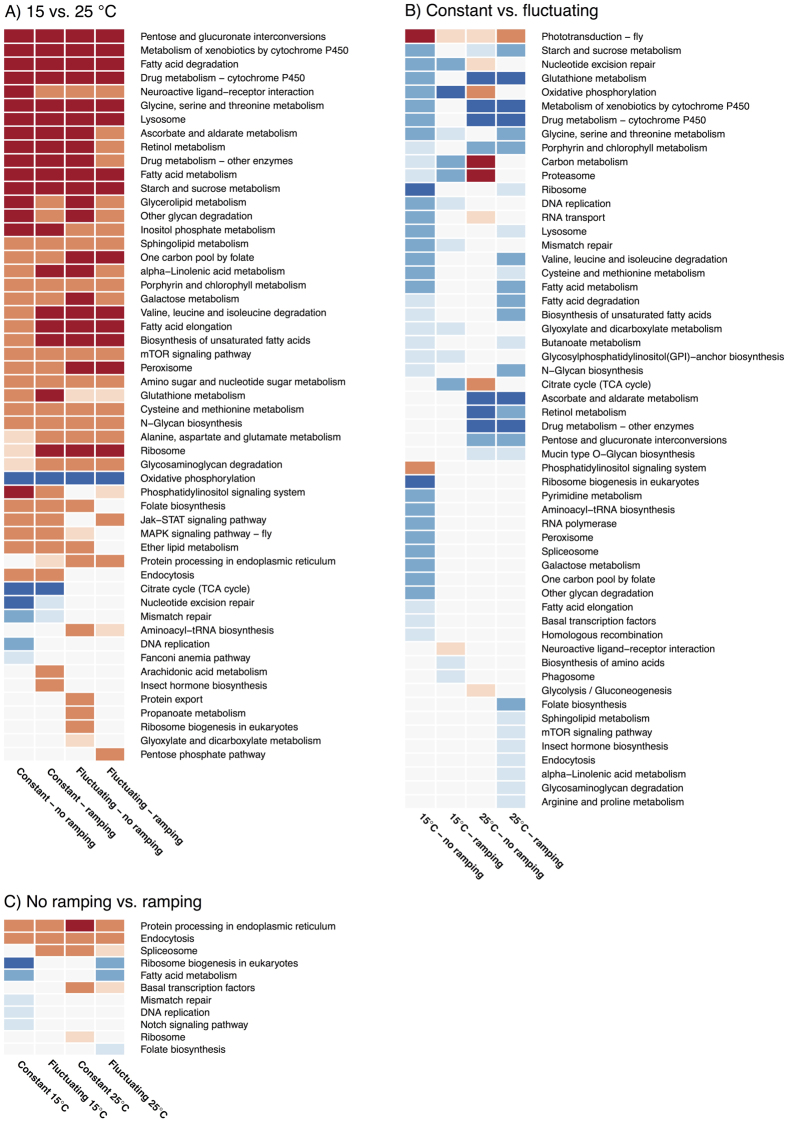
KEGG pathway analyses of the transcriptomic responses among main contrasts. KEGG pathway analyses were performed for the following three main contrasts: (**A**) Mean temperature (15 *vs.* 25 °C), (**B**) Temperature regime (constant *vs*. fluctuating) and (**C**) Thermal ramping (20 *vs.* 35 °C). In all panels, color signifies FDR adjusted significance (with progressively darker color associated with smaller P-values.) for a given KEGG pathway. Red color indicates up- and blue color indicates down-regulation at 25 °C, fluctuating temperature and ramping to 35 °C, respectively.

Gradually increasing the temperature to 35 °C led to a high and equal amount of up- and down-regulated genes (Fig. 3, Table S1). Few KEGG pathways were identified (Fig. 5C). The lack of response in the KEGG analysis reflects that the responding genes were not a part of the KEGG pathways rather than a lack of response. The relatively high amount of genes responding by up-regulation was shown by the gene ontology enrichment analysis to include stress responsive processes^24^ (Table S3). The pattern among the down-regulated genes was less clear and scattered among specific groups of functions not immediately related to the acute heat exposure (Table S3). As the general gene transcription and translation is suppressed during severe heat stress, the lack of a general metabolic suppression suggests that temperatures were not high enough to fully induce this suppression^24^.

### Correlation between gene expression and thermal tolerance

To identify candidate genes for heat tolerance we correlated gene expression across all genes with the heat tolerance phenotype at both 20 and at 35 °C. We found the expression of many genes to have a tight statistical association with heat tolerance. The majority of such genes showed a highly significant correlation at both temperatures, i.e. both before and during high temperature exposure. High expression of phototransduction genes and low expression of mitochondrial/energy production genes was associated with high temperature tolerance at both 20 and 35 °C. Further, a few chaperone genes and genes related to protein folding were associated at 35 but not at 20 °C suggesting that these genes contribute to short term acclimating (or hardening)^51^ during the heat exposure). Finally, seven genes were significantly correlated with heat tolerance, but negatively at 20 and positively at 35 °C. Among these several stress related genes (Hsp27, Cold shock domain-containing protein, Hsp90 Co-chaperone) were found. It is interesting that the expression at 20 °C so clearly predicts the resulting CTmax, underlining the importance of these genes for thermal tolerance. Moreover, the genes mostly predictive of heat tolerance showed a high degree of overlap with the genes affected by temperature fluctuations pointing to the potential importance of these genes and pathways identified for tolerance to this ecological relevant thermal regime.

### Overlaps with other studies on the transcriptomic basis of heat tolerance

Some inference about the mechanisms induced by the different treatments used here can be made by comparisons to other studies, although caution is warranted when comparing different genetic backgrounds^52^. As expected, heat ramping flies from 20 to 35 °C showed high overlap with genes earlier found to respond to short-term heat exposure (hardening)^24^. Genes that differed between constant and fluctuating regimes showed the largest association with genes found to respond to selection for increased heat knockdown time^53^, but was only weakly associated with genes earlier detected as heat responding (Figure S4). This underlines that genes induced by temperature fluctuations and by mean temperature represent separate and little explored mechanisms.

## Conclusion

The current knowledge of how organisms interact and respond to natural temperatures is primarily derived from studies on constant temperatures^23^. The results of this study of whole fly transcriptomics in a laboratory population suggest that the thermal acclimation of ectotherms is achieved by independent molecular pathways induced by mean and variation in temperature, in addition to the classical heat stress response. The response to mean temperatures is highly dependent on modification of a major part of the transcriptome (many genes), while the response to fluctuations is restricted to a much smaller set of genes in the transcriptomic response, including a strong signal from genes involved in environmental sensing (phototransduction). The independent acclimation mechanisms detected might explain why results from laboratory studies often do not match field findings^20^,^54^, and suggest that acclimation capacity is underestimated in laboratory studies that use constant temperatures^37^. Studies of the full performance curves could yield insight into the independent plastic responses to temperature mean and fluctuations and how they differ with respect to costs and benefits.

## Materials and Methods

### Experimental flies and thermal regimes

Males from a laboratory population of *D. melanogaster* founded from 589 isofemale lines collected in Denmark (2010) were used for this experiment^55^. Flies were reared from egg to adult and until six days of age in four different thermal regimes. Hereafter, the effects of the different thermal regimes were assessed by global gene expression analyses coupled with assays of upper thermal limits (Fig. 1). Experimental flies were density controlled^56^ by adding 30 eggs per 7 mL food vial. Egg collection was synchronized to ensure simultaneous emergence of experimental flies across temperature regimes (respective developmental times were estimated from pilot experiments). Each of the four temperature regimes was generated by a single custom build thermal cabinet at either constant 15 or 25 °C or fluctuating with mean temperatures of 15 or 25 °C, all with an identical 12:12 light:dark cycle. The fluctuating temperature regimes followed a Gaussian function yielding a daily fluctuation ranging from 12–20 °C and 22–30 °C, respectively (Fig. 1, Figure S1). The Gaussian model mimicked well the average daily variation in air and microhabitat temperatures measured at the site where flies were originally obtained suggesting that the model produced ecological relevant thermal fluctuations (Figure S1). Male flies were collected and pooled across 67 replicate vials every 7^th^ hour and maintained on fresh food with 20 flies per vial at the respective temperature regime. We used males to avoid confounding effects of reproduction status. Experimental flies from all treatments were six days old ±14 hours when assayed for either heat tolerance or transcriptomics. To ensure that all treatment groups experienced identical starting conditions for the thermal assays, flies were transferred to constant 20 °C eighteen hours prior to the start of the assay.

### Heat tolerance assay

The heat tolerance of male flies from the four temperature treatments was measured as CTmax and determined by a heat ramping assay^33^. All flies from all treatments were assayed in a single run. Flies (47–50 replicate flies per treatment) were quickly loaded individually into 5 mL screw-cap glass vials and placed in racks in a temperature controlled glass water tank. The temperature was ramped up from 20 °C at a rate of 0.1 °C min^−1^ until all flies were immobile. Flies were continuously monitored, and the temperature where flies stopped moving any body part was registered for each fly individually.

### Transcriptomics (RNA extraction and array hybridization)

For transcriptomic studies male flies from all four experimental treatments were transferred into four replicate 5 mL plastic vials in groups of 20 per vial. Samples were maintained in parallel at 20 °C (in a control water bath) or ramped from 20 °C at a rate of 0.1 °C min^−1^ until 35 °C in the water bath where the heat tolerance assay took place. Thus, flies assayed for heat tolerance and for transcriptomics were from the same generation and exposed to increasing temperature in the same water bath at the same time. When the temperature of the heat ramping assay reached 35 °C, the samples destined for transcriptomics (i.e. all samples from both 20 and 35 °C treatments) were simultaneously snap frozen in liquid nitrogen (Fig. 1). In total, 24 microarray experiments (four developmental treatments x two heat ramping treatments x three biological replicates) were performed for this study by AROS Applied Biotechnology A/S, Aarhus, Denmark. The Affymetrix Drosophila Genome 2.0 Array used here contains 18,880 probes analysing over 18,500 transcripts covering the vast majority of known and predicted genes. The RNA extraction and creation of cDNA were performed as described in Dyrskjøt, *et al*.^57^. Fifteen micrograms of cDNA were fragmented and loaded onto the Affymetrix probe array cartridge. Incubation, washing and staining procedures were performed in the Affymetrix Fluidics Station 450. The probe arrays were scanned at 560 nm using a confocal laser-scanning microscope (Affymetrix Scanner 3000 7G; Affymetrix, Santa Clara, CA, USA). The raw data was GC-RMA normalized^58^ using the gcrma package (v. 2.40.0) in R.

### Statistical Analysis

Heat tolerance data was analysed for effects of mean temperature (15 or 25 °C) and variability of temperature (constant or fluctuating) using a two-way ANOVA.

Differential expression of individual genes within each contrast was assessed using linear modeling and empirical Bayes methods in the Limma package^59^ (version 2.18.2) in R using FDR adjustment of the P-values. The empirical P-value for the observed overlap of genes among the different treatments was determined using Monte Carlo simulations. In each simulation, the gene list for each treatment was permutated (100,000 times) and the random overlap among gene lists was recorded. The empirical P-value was determined as the fraction of all permutations where the random overlap was larger or equal to the observed overlap among the gene lists.

We used the “unpaired” method implemented in GAGE^34^ to detect significantly differentially regulated KEGG pathways^60^,^61^ for each treatment contrast. For the contrasts within temperature regime (fluctuating *vs*. constant) and thermal ramping (35 *vs.* 20 °C) we used a significance threshold of FDR adjusted P-values at 0.05, while we used a significance threshold of 0.01 for mean developmental temperature (25 *vs.* 15 °C). Gene sets from the individual contrasts were also analysed based on GO terms created by the biomaRt package^62^ (version 2.24.0) and analysed by the PIANO (Platform for Integrative Analysis of Omics data) package^63^ (version 1.8.2) in R. Gene set analysis (GSA) in PIANO identified the significant enrichment of GO terms (restricted to minimum 10 and maximum 200 genes) based on the Limma P-values for all genes in each contrast. Significance for each GO term was evaluated based on a FDR adjusted P-value cutoff of 0.005 using 10^6^ permutations.

Gene expression of all samples from 20 °C (n = 12) and all samples from 35 °C (n = 12) was further correlated to the respective heat tolerance of the appropriate treatment group (four treatment groups). To compensate for the limited number of data points in this exploratory correlation we used a conservative significance threshold of P < 0.001. Functional annotation clustering of genes correlated to the heat tolerance phenotype was performed using the DAVID database^64^ to establish enrichment of functional groups of genes.

### Data Availability

Phenotypic heat tolerance data is deposited in Dryad (doi: 10.5061/dryad.571f8). Microarray gene expression data is deposited to the Gene Expression Omnibus database (GSE84680).

## Additional Information

**How to cite this article**: Sørensen, J. G. *et al*. Thermal fluctuations affect the transcriptome through mechanisms independent of average temperature. *Sci. Rep.* **6**, 30975; doi: 10.1038/srep30975 (2016).

## Supplementary Material

Supplementary Information

Supplementary Table S1

Supplementary Table S2

Supplementary Table S3

## Figures and Tables

**Table 1:** Three-way ANOVA of PCA of gene expression.

Source of Variation (df)	PC1 (32.1%)	PC2 (15.6%)	PC3 (8.1%)	PC4 (6.5%)
	F	F	F	F
Mean (1, 16)	281.8***	3.8	0.003	1.4
Fluctuation (1, 16)	5.3*	0.3	2.4	23.3***
Ramping (1, 16)	1.1	771.0***	0.6	0.04
Mean × Fluctuation (1, 16)	4.2	7.1*	8.1*	0.8
Mean × Ramping (1, 16)	0.2	0.2	5.2*	0.1
Fluctuation × Ramping (1, 16)	0.04	7.4E−04	0.1	0.02
Mean × Fluctuation × Ramping (1, 16)	1.7	0.5	0.006	2.1

The results of the analysis of the first four PCs are given with the percentage of variance explained in brackets. Mean temperature (Mean) represents mean developmental temperatures of either 15 °C or 25 °C, Fluctuating environment (Fluctuation) represents development at either constant or fluctuating temperatures, and adult high temperature treatment (Ramping) represents either controls kept at 20 °C or ramped to 35 °C at a rate of 0.1 °C min^−1^. *P < 0.05, ***P < 0.001.

**Table 2:** Functional annotation enrichment analyses of genes with significant correlation between gene expression and heat resistance.

Positively correlated at 35 and 20 °C (n = 564)	Negatively correlated at 35 and 20 °C (n = 828)	Correlated at 35 but not at 20 °C (n = 237)
Detection of light stimulus (0009583)	Generation of precursor metabolites and energy (0006091)	Protein folding (0006457)
Phototransduction (0007602)	Mitochondrial membrane part (0044455)	Chaperonin-containing T-complex (0005832)
Response to light stimulus (0009416)	Cellular respiration (0045333)	Unfolded protein binding (0051082)
Detection of abiotic stimulus (0009582)	Organelle inner membrane (0019866)	mRNA metabolic process (0016071)
Detection of external stimulus (0009581)	Mitochondrial inner membrane (0005743)	Nucleoplasm (0005654)
Visual perception (0007601)	Oxidative phosphorylation (0006119)	Posttranscriptional regulation of gene expression (0010608)

The CTmax of each acclimation regime was correlated to the expression of each gene of the same regime prior to ramping (at 20 °C), as well as to the gene expression at the final stage of ramping (at 35 °C). Only genes with expression significantly correlated to CTmax at either 20 and/or 35 °C were included in the analysis. Here the results are truncated to represent the top six enriched GO terms in each category. See Table S4 for full list of clusters including all processes and the associated data-bases, number of genes, raw and corrected P-values. GO-term ID is presented in parenthesis after the GO-term.
