# Supplementary material for: Thermal fluctuations affect the transcriptome through mechanisms independent of average temperature
Source: Sci Rep. 2016 Aug 4;6:30975. doi: 10.1038/srep30975 (PMC4973280; doi:10.1038/srep30975)
Supplement: Supplementary Information [file srep30975-s1.pdf]

Thermal fluctuations affect the transcriptome through mechanisms independent of average temperature

Jesper Givskov Sørensen[1*], Mads Fristrup Schou[1], Torsten Nygaard Kristensen[2], and Volker Loeschcke[1]

1: Department of Bioscience, Section for Genetics, Ecology and Evolution, Aarhus University, Ny Munkegade 114, 8000 Aarhus C, Denmark

2: Department of Chemistry and Bioscience, Aalborg University, Fredrik Bajers Vej 7H, 9220 Aalborg E, Denmark

***Correspondence: Jesper G Sørensen; jesper.soerensen@bios.au.dk; phone +45 30183160**

Supplemental material:

Table S1. Table shows all significant gene lists in individual contrasts and lists of genes shared among individual contrasts within main contrasts.

Table S2. Table shows all individual two-way overlaps among individual contrasts, both within and between main contrasts.

Table S3. Table shows all significant enriched GO terms from the PIANO gene set analyses ($P < 0.005$).

Table S4. Table shows results of the correlation of gene expression with thermal tolerance phenotype for all genes and corresponding DAVID (http://david.abcc.ncifcrf.gov/) functional annotation enrichment analyses of significant gene lists. The table provides all statistics of the correlations including effect size.

Figure S1. Natural and laboratory temperatures relevant for the study. A) Natural temperatures occurring in the microhabitats where the population used in this study was originally collected. Data loggers (iButton® Data Loggers, Maxim, Sunnyvale, California, USA) were used to register temperatures at the collection site (a heap of discarded apples at a fruit orchard) every 10 minutes data modified from [52]. The temperatures shown were collected during one month in spring (15th March to 16th April 2014) and present shaded air temperature (1 meter above ground) and at the surface of the apple heap. It is obvious from the graph that the apple heap microhabitat has buffered temperature fluctuations compared to air temperatures. Light areas on the graph correspond to day periods (8 AM to 8 PM), and dark areas correspond to night time (8 PM to 8 AM). B) Daily average temperature fluctuations in the apple heap (data from A). C) Daily average temperature fluctuations in the air (data from A). Data of B and C was centered to a mean temperature of 0 within each day for comparison. D) Actual temperatures experienced by flies in the thermal treatments. Data given here shows mean (± SD) across eight consecutive days during the experiment. The figure shows that temperatures were accurately controlled by the thermal cabinets. The temperature profile followed a Gaussian function, which reasonably

mimics the expected thermal fluctuations in the natural environment of the original population of flies (compare B, C, D).

Figure S2. The number of significant genes differentially expressed among treatments. Genes with adjusted P-values (P adj.) in the Limma analysis < 0.05 were considered significant. Two main variables were considered: Mean temperature (15 or 25 °C) or fluctuation (constant or fluctuating temperature). See Figure 1 for details of the experimental design. Numbers in the Venn diagrams represent shared genes among individual contrasts within main treatments [Panel A: Fluctuation (Constant *vs*. fluctuating temperature); B: Mean temperature (15 *vs*. 25 °C)]. Numbers in brackets signify genes shared among all three contrasts.

Figure S3. Vulcano plots showing the log2 fold change against the –log10(P-value) for each transcript in each of the four individual contrasts within A) Mean temperature (15 *vs*. 25 °C), B) Fluctuation (Constant *vs*. fluctuating temperature), and C) Ramping (No ramping (20 °C) *vs*. ramping (35 °C)).

Figure S4. Table show number of genes from this study overlapping with significant gene lists from two other transcriptomics studies on heat adaptation and responses to heat exposure. (1) Sørensen, et al. [50], where the response in gene expression to selection was assayed. Selection regimes imposed were development (egg to pupae) at constant 30 °C (C30; 225 genes), survival to a heat shock (HS; 911 genes) and knockdown time (KD; 399 genes) when exposed to high temperature. (2) Sørensen, et al. [28], where the time dependent response to a sub-lethal heat shock was assayed. Here, three main clusters of heat responsive genes were identified as responding either early up (265 genes), early down (505) or late up (226 genes). Both these studies investigated gene expression in female flies. Green color signifies a significant probability (P < 0.01) for observing the observed overlap by chance, given the respective gene lists compared, with progressively darker green associated with smaller P-values.

Supplementary Table S2. Overlap among all individual treatments including the probability of obtaining this overlap by chance based on the number of significant genes in each contrast (on the diagonal of panel A). Contrasts are defined by the following codes and colours: Yellow: Thermal ramping (20 *vs.* 35 °C); Red: Temperature regime (constant *vs.* fluctuating); Blue: Mean temperature (15 *vs.* 25 °C).

		Ramping (20 vs 35 °C)			*Fluctuation of environment (fluctuating vs constant)*				*Developmental accl. (15 vs 25 °C)*			
Overlap # genes	***15°C Con***	***15°C Fluc***	***25°C Con***	***25°C Fluc***	***15°C@20°C***	***15°C@35°C***	***25°C@20°C***	***25°C@35°C***	***Con@20°C***	***Con@35°C***	***Fluc@20°C***	***Fluc@35°C***
15°C Con	2564	1731	1781	1848	85	128	52	48	1400	1518	1211	1060
15°C Fluc		2843	1899	1894	83	148	56	53	1538	1671	1306	1190
25°C Con			3296	2271	133	163	59	63	1821	1940	1465	1345
25°C Fluc				2996	78	113	64	51	1590	1743	1379	1247
15°C@20°C					348	146	5	24	292	201	124	115
15°C@35°C						462	3	24	343	320	163	153
25°C@20°C							154	33	112	96	96	82
25°C@35°C								189	138	142	96	110
Con@20°C									7890	6312	5133	4788
Con@35°C										8084	5444	5251
Fluc@20°C											6501	4762
Fluc@35°C												6350

P-value (overlap)	***15°C Con***	***15°C Fluc***	***25°C Con***	***25°C Fluc***	***15°C@20°C***	***15°C@35°C***	***25°C@20°C***	***25°C@35°C***	***Con@20°C***	***Con@35°C***	***Fluc@20°C***	***Fluc@35°C***
15°C Con		< 1E-6	< 1E-6	< 1E-6	< 1E-6	< 1E-6	< 1E-6	2.0E-5	< 1E-6	< 1E-6	< 1E-6	< 1E-6
15°C Fluc			< 1E-6	< 1E-6	< 1E-6	< 1E-6	< 1E-6	< 1E-6	< 1E-6	< 1E-6	< 1E-6	< 1E-6
25°C Con				< 1E-6	< 1E-6	< 1E-6	< 1E-6	< 1E-6	< 1E-6	< 1E-6	< 1E-6	< 1E-6
25°C Fluc					4.2E-4	< 1E-6	< 1E-6	2.0E-5	< 1E-6	< 1E-6	< 1E-6	< 1E-6
15°C@20°C						< 1E-6	0.07	< 1E-6	< 1E-6	< 1E-6	0.28	0.55
15°C@35°C							0.52	< 1E-6	< 1E-6	< 1E-6	0.31	0.55
25°C@20°C								< 1E-6	< 1E-6	< 1E-6	< 1E-6	< 1E-6
25°C@35°C									< 1E-6	< 1E-6	< 1E-6	< 1E-6
Con@20°C										< 1E-6	< 1E-6	< 1E-6
Con@35°C											< 1E-6	< 1E-6
Fluc@20°C												< 1E-6
Fluc@35°C												

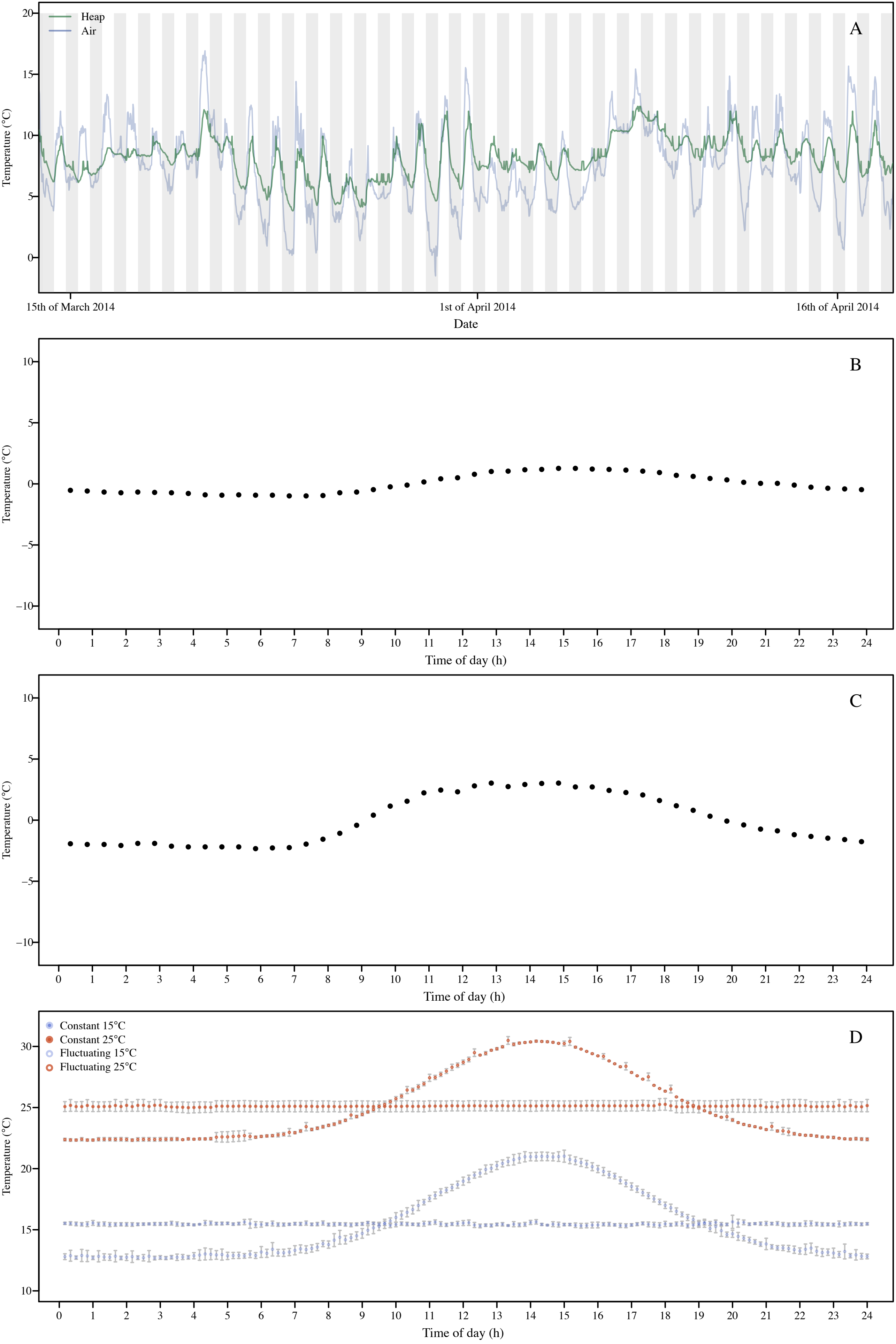

Heap
Air
A
Temperature (°C)
15th of March 2014
1st of April 2014
16th of April 2014
Date
B
Time of day (h)
C
D
Constant 15°C
Constant 25°C
Fluctuating 15°C
Fluctuating 25°C

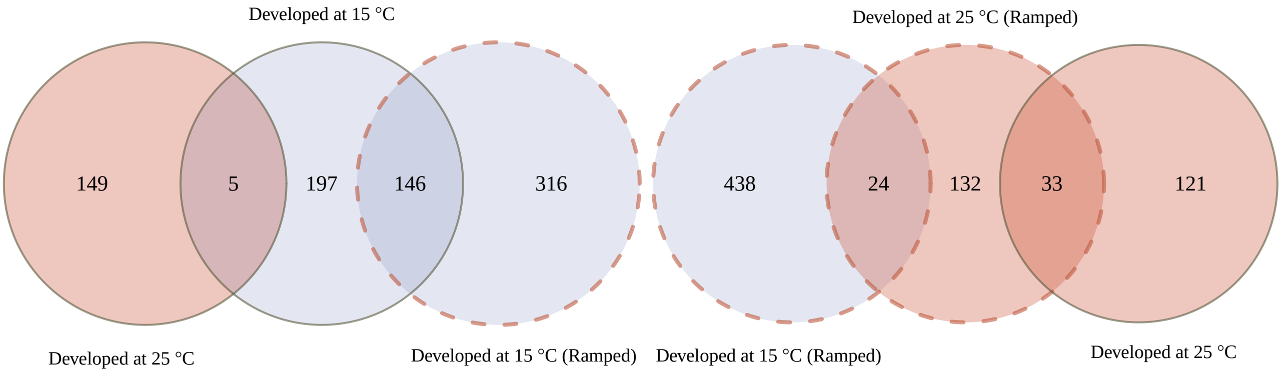
Developed at 15 °C
Developed at 25 °C (Ramped)
149
5
197
146
316
438
24
132
33
121
Developed at 25 °C
Developed at 15 °C (Ramped)
Developed at 15 °C (Ramped)
Developed at 25 °C

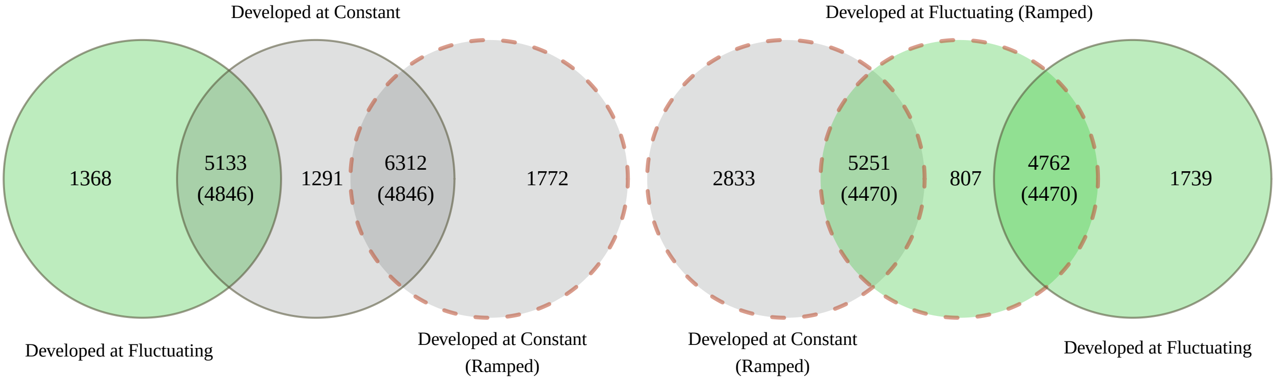
Developed at Constant
1368
5133
(4846)
1291
6312
(4846)
1772
Developed at Fluctuating
Developed at Constant (Ramped)
Developed at Fluctuating (Ramped)
2833
5251
(4470)
807
4762
(4470)
1739
Developed at Constant (Ramped)
Developed at Fluctuating

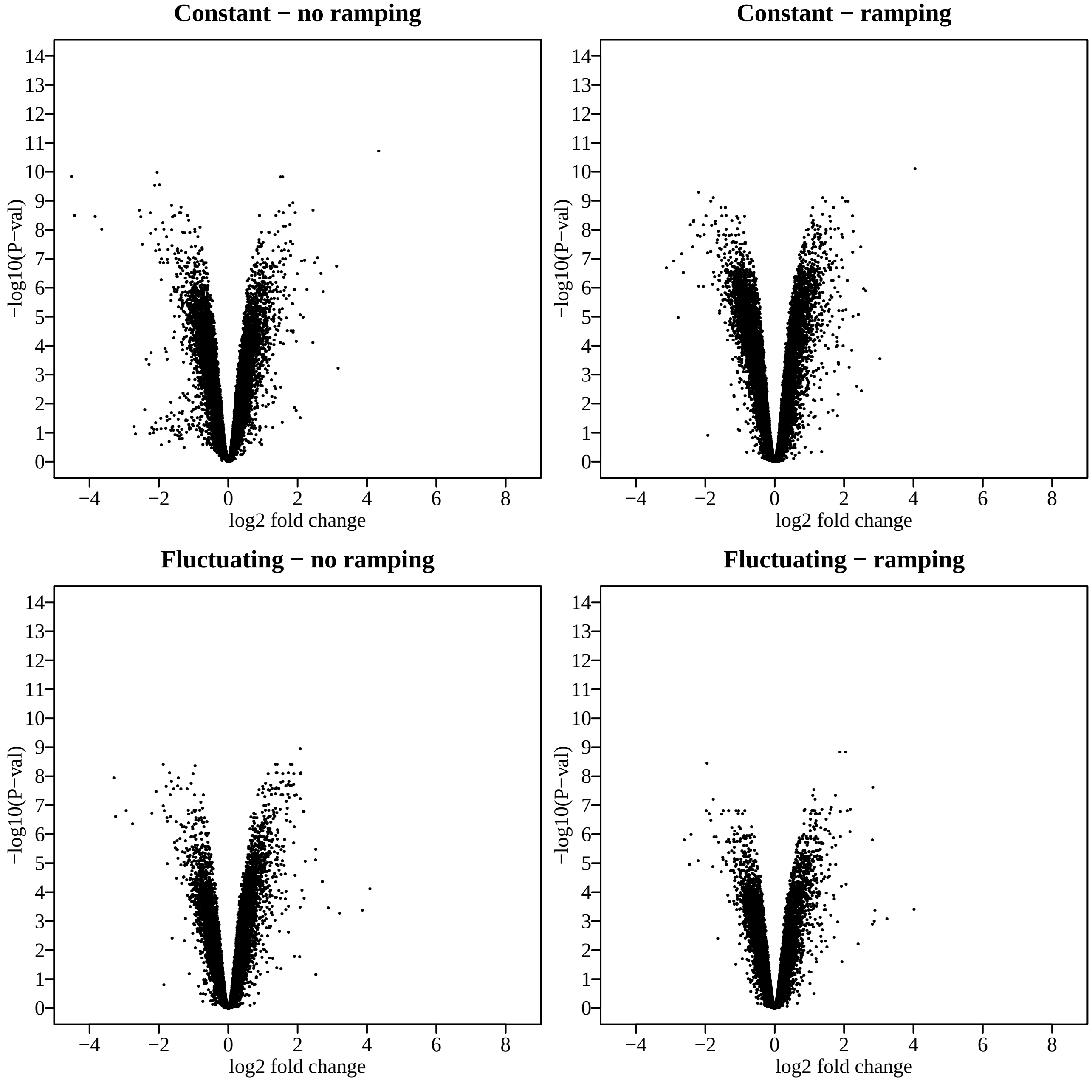
Constant − no ramping
Constant − ramping
Fluctuating − no ramping
Fluctuating − ramping
−log10(P−val)
log2 fold change
0
1
2
3
4
5
6
7
8
9
10
11
12
13
14
−4
−2
0
2
4
6
8

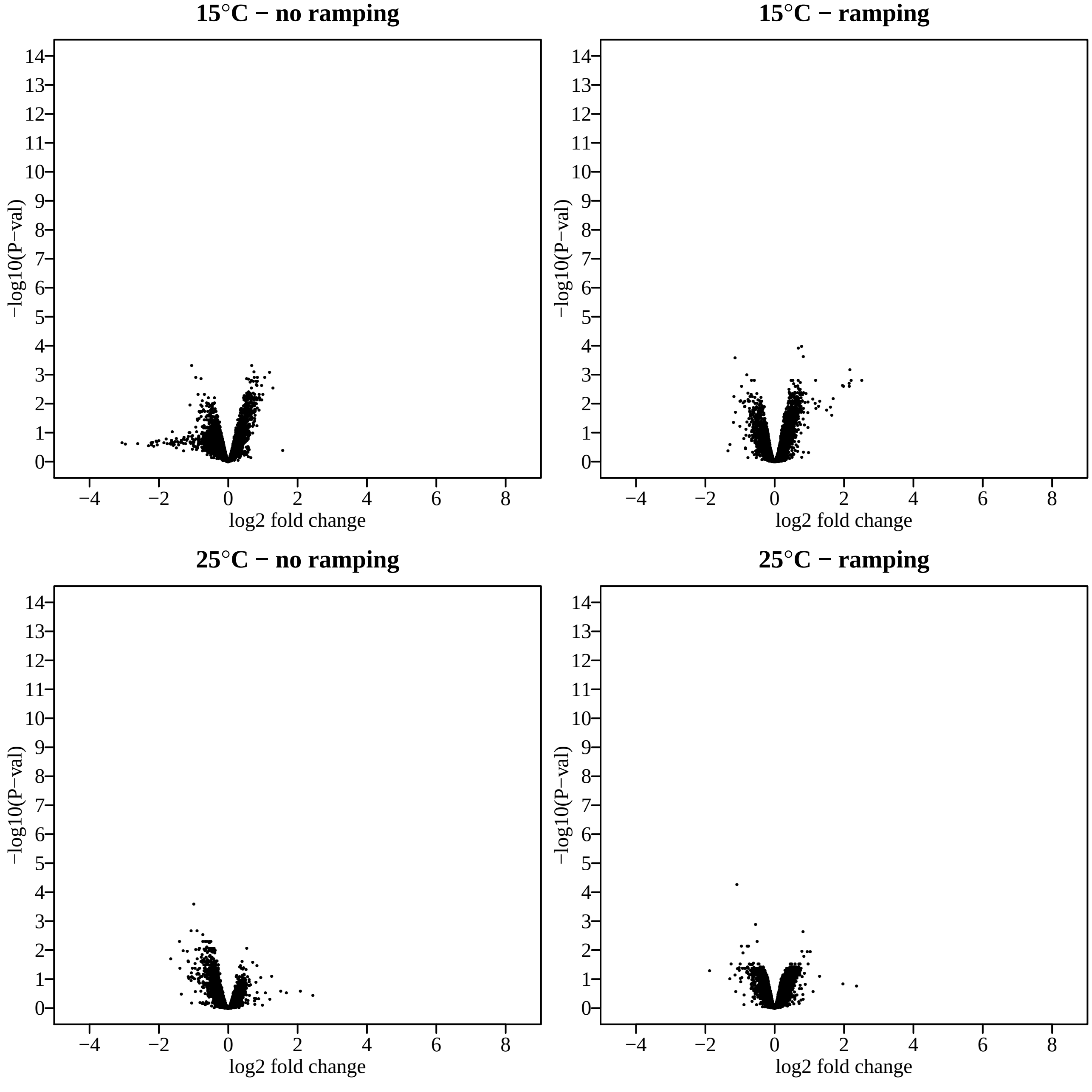
15°C − no ramping
15°C − ramping
25°C − no ramping
25°C − ramping
−log10(P−val)
log2 fold change
0
1
2
3
4
5
6
7
8
9
10
11
12
13
14
−4
−2
0
2
4
6
8

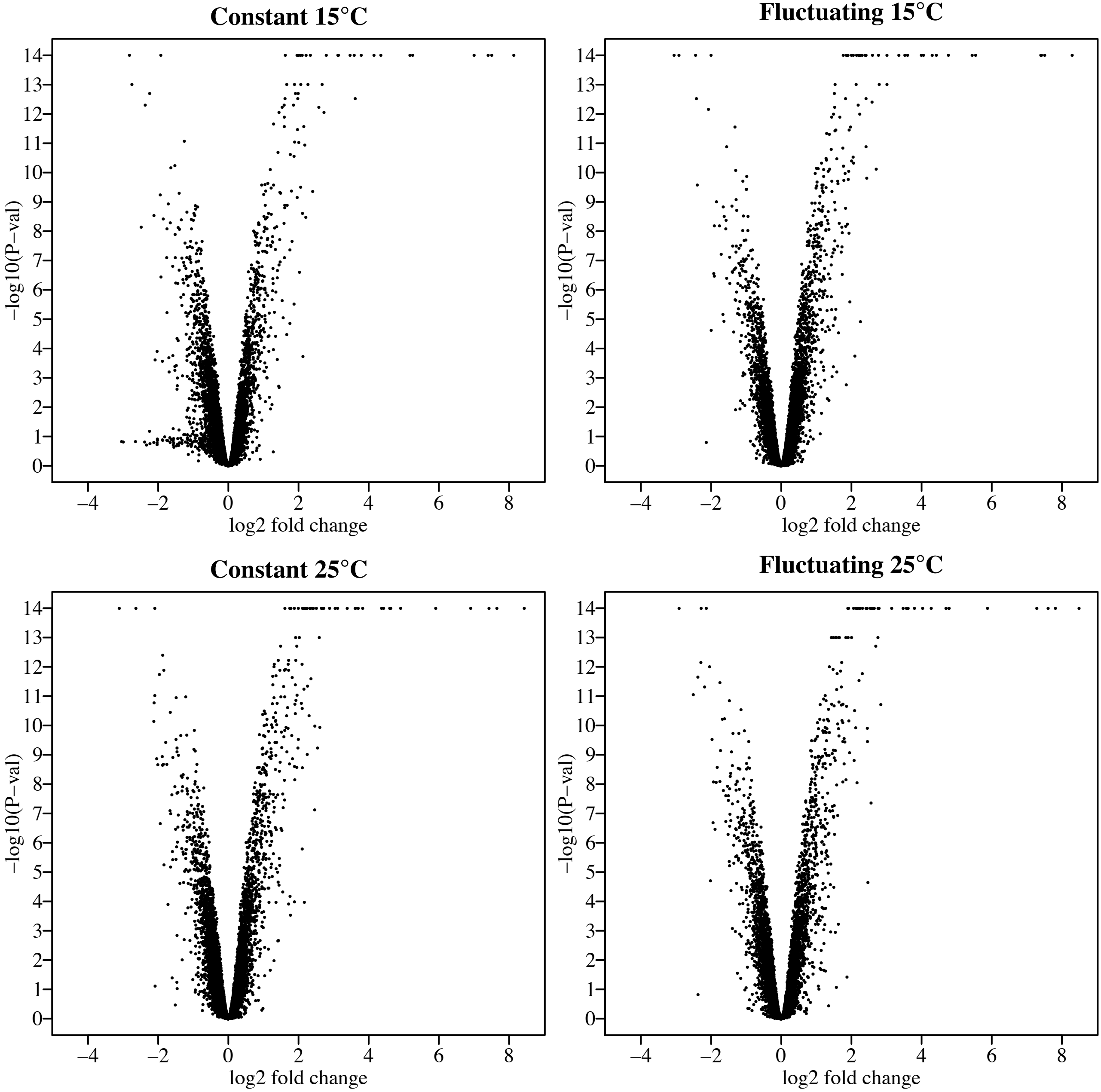
Constant 15°C
Fluctuating 15°C
Constant 25°C
Fluctuating 25°C
−log10(P−val)
log2 fold change

		Selection for			Heat responding		
		C30	HS	KD	Early down	Early up	Late up
Adult thermal treatment	Constant (15°C)	54	198	106	177	124	77
	Fluctuating (15°C)	53	216	100	177	133	77
	Constant (25°C)	54	245	104	175	135	93
	Fluctuating (25°C)	57	210	104	176	129	78
Fluctuation	15°C	14	40	30	14	3	9
	15°C (Ramped)	11	54	24	12	7	12
	25°C	2	10	10	4	5	6
	25°C (Ramped)	4	16	18	10	9	2
Mean temperature	Constant	117	519	217	341	145	149
	Constant (Ramped)	117	512	211	346	159	142
	Fluctuating	91	423	182	342	108	125
	Fluctuating (Ramped)	87	384	160	313	93	109
